# Rivaroxaban Once-Daily vs. Dose-Adjusted Vitamin K Antagonist on Biomarkers in Acute Decompensated Heart Failure and Atrial Fibrillation (ROAD HF-AF): Rationale and Design of an Investigator-Initiated Multicenter Randomized Prospective Open-Labeled Pilot Clinical Study

**DOI:** 10.3389/fcvm.2021.765081

**Published:** 2022-01-12

**Authors:** Iksung Cho, Jaewon Oh, In-Cheol Kim, Hyemoon Chung, Jung-Hee Lee, Hyue Mee Kim, Young Sup Byun, Byung-Su Yoo, Eui-Young Choi, Wook-Jin Chung, Wook Bum Pyun, Seok-Min Kang

**Affiliations:** ^1^Cardiology Division, Cardiovascular Research Institute, Severance Cardiovascular Hospital, Yonsei University College of Medicine, Seoul, South Korea; ^2^Division of Cardiology, Keimyung University Dongsan Medical Center, Daegu, South Korea; ^3^Division of Cardiology, Department of Internal Medicine, Kyung Hee University, Seoul, South Korea; ^4^Division of Cardiology, Department of Internal Medicine, Yeungnam University Medical Center, Daegu, South Korea; ^5^Division of Cardiology, Department of Internal Medicine, Chung-Ang University, Seoul, South Korea; ^6^Division of Cardiology, Sanggye-Paik Hospital, Inje University, Seoul, South Korea; ^7^Department of Internal Medicine, Yonsei University Wonju College of Medicine, Wonju, South Korea; ^8^Division of Cardiology, Heart Center, Gangnam Severance Hospital, Yonsei University College of Medicine, Seoul, South Korea; ^9^Department of Cardiovascular Medicine, Gachon University Gil Medical Center, Incheon, South Korea; ^10^Division of Cardiology, Department of Internal Medicine, Ewha Womans University, Seoul, South Korea

**Keywords:** rivaroxaban, acute decompensated heart failure, atrial fibrillation, vitamin K antagonist (VKA), biomarker

## Abstract

**Background:** Clinical trials of non-vitamin K antagonist oral anticoagulants (NOACs) in patients with chronic heart failure and atrial fibrillation (AF) have demonstrated reduced risks of stroke and bleeding compared with vitamin K antagonists (VKAs). Here, we aim to assess the clinical efficacy and safety of rivaroxaban, a NOAC, compared with warfarin, a VKA, and the effects of rivaroxaban on cardiovascular biomarkers in patients with acute decompensated heart failure (ADHF) with reduced ejection fraction (≤40%) and AF.

**Methods:** Rivaroxaban Once-daily vs. dose-adjusted vitamin K antagonist on biomarkers in Acute Decompensated Heart Failure and Atrial Fibrillation (ROAD HF-AF) is a randomized, open-labeled, controlled, prospective, multicenter pilot study designed to assess cardiovascular biomarkers and the safety of rivaroxaban (20 or 15 mg in patients with creatinine clearance 30–49 mL/min per day) compared with VKA (target international normalized range: 2–3) in 150 patients hospitalized with ADHF and AF. The primary endpoint is the change in circulating high-sensitivity cardiac troponin (hsTn) during hospitalization. The secondary endpoints are bleeding, hospital stay duration, in-hospital mortality, and changes in cardiovascular, renal, and thrombosis biomarkers. Patients will be followed for 180 days.

**Conclusion:** We hypothesize that rivaroxaban will reduce myocardial injury and hemodynamic stress, as reflected by the biomarker status, within 72 h in patients with ADHF and AF, compared with VKA. We hope to facilitate future biomarker-based, large-scale outcome trials using NOACs in patients with ADHF and AF, based on the results of this multicenter, randomized, controlled study.

## Introduction

The prevalence of heart failure (HF) is rapidly increasing and is the leading cause of hospitalization in people aged over 65 years in developed countries (1, 2). Acute decompensated heart failure (ADHF) is a significant public health issue due to the substantial morbidity and mortality rates, including a high hospital readmission rate. Hypercoagulability is suggested as a risk factor for poor outcomes in patients with ADHF. For example, in a community-based study in the United States, ischemic stroke incidence was significantly higher in patients with HF than in the general population in the first 30 days after HF diagnosis and remained high during a 5-year follow-up (3). The prevalence of atrial fibrillation (AF) in patients with HF is high, and AF is the main factor driving the high incidence of thromboembolic events (4). Furthermore, comorbidities and factors that increase the risk of thromboembolic events in patients with AF, including old age, coronary artery disease, hypertension, and diabetes, are common in patients with both compensated and decompensated HF (5). Therefore, optimal anticoagulation is a potential strategy to improve outcomes in patients with ADHF.

Traditionally, vitamin K antagonists (VKAs), such as warfarin, are recommended to reduce thromboembolic event risk in patients with AF and HF. However, VKAs are limited by their interactions with other drugs and diet. Importantly, drug levels are influenced by the worsening renal function, liver congestion, and hemodynamic alterations observed in patients with ADHF. Non-vitamin K antagonist oral anticoagulants (NOACs), including rivaroxaban, apixaban, dabigatran, and edoxaban, are alternatives to VKAs, and they have demonstrated improved efficacy for stroke prevention and safety compared with VKAs in patients with HF and AF (6, 7). The benefits of NOACs compared with VKAs include fewer food and drug interactions and fixed dosing. Recently, the inhibition of thrombin generation has been suggested as a potential benefit of NOACs, especially rivaroxaban (8, 9). Thrombin is a key component in the coagulation pathway, and it enhances platelet activation and aggregation; thus, direct inhibition of the common pathway by antithrombotic therapy may mitigate ongoing myocardial injury in patients with ADHF (9). A *post-hoc* analysis of the COMMANDER HF trial demonstrated that 2.5 mg rivaroxaban twice daily significantly reduced stroke or transient ischemic attack (TIA) rate compared with placebo in chronic HF patients with coronary artery disease and sinus rhythm following recent worsening episodes (10). However, the clinical efficacy and safety of rivaroxaban in patients with ADHF and AF are unknown.

In patients with ADHF, biomarker analysis is utilized for accurate diagnosis and prognostication. Furthermore, biomarkers can provide valuable information on the pathophysiology of ADHF and the mechanism underlying the treatment effects (11). Therefore, it would be reasonable and informative to assess the potential benefits of rivaroxaban on myocardial and renal damage compared with VKAs, using surrogate biomarkers in hospitalized patients with ADHF and AF, in addition to the clinical efficacy and safety outcomes. In patients with ADHF, numerous cardiovascular biomarkers can be used to reflect hemodynamic stress and myocardial injury resulting from inflammation, neurohormonal and endothelial dysfunction, and microvascular thrombosis (11). Recently, high-sensitive troponin (hsTn) has emerged as a novel prognostic marker in patients with ADHF (12). Coronary microvascular dysfunction has been reported to be correlated with hsTn in patients with non-ischemic HF (13). In a sub-study of the RELAX-AHF trial, an increase in hsTn was related to poor clinical outcomes in patients with ADHF (14). Additionally, *post-hoc* analyses of the Val-HeFT and GISSI-HF trials demonstrated the clinical importance of ongoing myocardial injury in patients with HF (15). Specifically, increased plasma hsTn level was predictive of an increased risk of all-cause mortality in patients with HF. Therefore, ongoing myocardial injury, reflected by elevated plasma hsTn level, is a substantial risk factor for patients with ADHF, and this may be related to microvascular thrombosis. However, evidence demonstrating the benefit of antithrombotic therapy with direct factor Xa inhibition in patients with ADHF is lacking. Therefore, in the Rivaroxaban Once-daily vs. dose-adjusted vitamin K antagonist on the biomarkers in Acute Decompensated Heart Failure and Atrial Fibrillation (ROAD HF-AF) study, we will examine the potential beneficial effects of rivaroxaban vs. warfarin in patients with ADHF with reduced ejection fraction (EF) and AF.

## Study Design

### Overall Design and Ethics Approval

**Figure 1:**
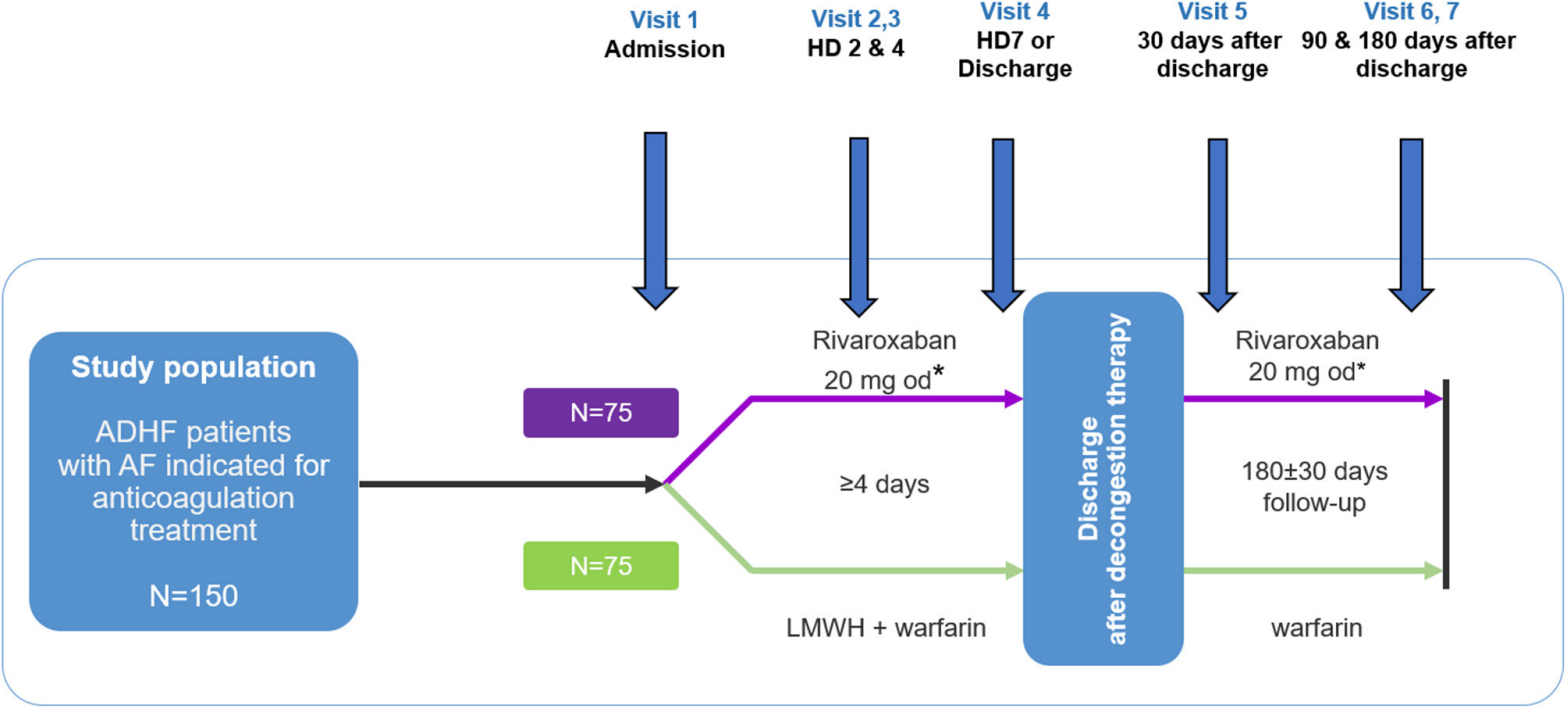
Study design. ADHF, acute decompensated heart failure; HD, hospital day; LMWH, low molecular weight heparin. ^*^Patients with an estimated glomerular filtration rate using the chronic kidney disease epidemiology collaboration (CKD-EPI) equation of 30–49 mL/min/1.73 m^2^ will receive 15 mg once daily.

ROAD HF-AF is a prospective, multicenter, randomized, parallel-group, open-label exploratory study designed to assess the efficacy and safety of rivaroxaban compared with warfarin using the change in surrogate biomarkers in patients with ADHF and AF (Figure 1). Patients will be followed during and after hospitalization for 6 months. The study will be conducted in accordance with the International Conference on Harmonization Good Clinical Practice guidelines and principles in the Declaration of Helsinki. Written informed consent will be obtained from patients before study entry. The study has been approved by the institutional review board of each participating center (No. 4-2017-0776). The study is sponsored by Bayer Pharma (Berlin, Germany) and registered at clinicaltrials.gov (NCT03490994).

### Objectives and Endpoints

The primary study objective is the maximum change in the hsTn level over 72 h from admission following treatment (Table 1). The maximum change in hsTn is defined as the greatest change from the natural log-transformed baseline hsTn value to the natural log-transformed peak hsTn value during hospitalization. The secondary objectives are the: (1) change in the hsTn level 30 and 180 days after treatment with rivaroxaban or warfarin [target international normalized ratio (INR): 2–3]; (2) change in D-dimer level during and after hospitalization as a thrombogenicity marker; (3) change in other biomarkers of cardiac fibrosis [soluble ST2 (sST2) and galectin-3], renal injury [cystatin C, neutrophil gelatinase-associated lipocalin (NGAL), and N-acetyl-β-D-glucosaminidase (NAG)], and thrombogenicity [thrombin–antithrombin (TAT) complex and plasminogen activator inhibitor type 1 (PAI-1)], hs-CRP, and NT-proBNP during and after hospitalization; (4) incidence and rate of major bleeding according to the International Society on Thrombosis and Haemostasis criteria (16) (e.g., bleeding causing a decrease in hemoglobin level of ≥2 g/dL; bleeding leading to transfusion; symptomatic bleeding in critical areas including intracranial, intraspinal, intraocular, pericardial, intraarticular, intramuscular with compartment syndrome, and retroperitoneal; and fatal bleeding causing death) or minor bleeding event during and after hospitalization; (5) hospital stay duration at initial hospitalization; (6) incidence of all-cause mortality during hospitalization and at 180-day follow-up; and (7) incidence of all-cause mortality and rehospitalization during hospitalization and at 180-day follow-up.

**Table 1:** Study endpoints.

**Primary endpoint**
• Change in hsTn over 72 h from admission after treated with rivaroxaban or warfarin
**Secondary endpoint**
• Change in hsTn from the baseline following hospitalization and 30 and 180 days after treatment with rivaroxaban or warfarin • Change in D-dimer from the baseline during and after hospitalization (Days 2, 4, and 7 or discharge, and 30 and 180 days after discharge) • Change in other biomarkers, including TAT complex, PAI-1, hs-CRP, NT-proBNP, sST2, galectin-3, cystatin C, NGAL, and NAG from baseline during (Day 7 or discharge) and after hospitalization (30 and 180 days after discharge) • Incidence and rate of major/minor bleeding events during and after hospitalization • Length of hospital stay at initial hospitalization • Incidence of all-cause mortality during and after hospitalization • Incidence of all-cause mortality and rehospitalization during the 180-day follow-up

*hsTn, high-sensitive troponin; TAT, thrombin–antithrombin complex; PAI-1, plasminogen activator inhibitor type 1; hs-CRP, high-sensitivity C-reactive protein; NT-proBNP, N-terminal proBNP; sST2, soluble ST2; NGAL, neutrophil gelatinase-associated lipocalin; NAG, N-acetyl-β-D-glucosaminidase*.

### Data Monitoring and Study Management

A Data Safety Monitoring Committee (DSMB) composed of independent experts, will be responsible for overseeing patient safety. Study sites will be randomly monitored at least once a year. During site visits, the monitors will review protocol compliance to ensure that data are obtained for all eligible patients and verify source documents. All clinical events, including hospitalizations and deaths, will be monitored and verified by an adjudication committee, composed of independent experts. The adjudication committee will be composed of 2 independent experts and one chairperson. A disagreement will be reviewed by the two reviewers and tried to be resolved. If the two adjudicators disagree, the chairperson will receive the material together with both proposals and will select one proposal, overruling the other (17).

### Participants

The study population will comprise ADHF patients with AF who have reduced EF and are hospitalized with a primary diagnosis of ADHF. The eligible patients at 10 participating hospitals in South Korea who meet all eligibility criteria will be considered for enrolment. Detailed inclusion and exclusion criteria are shown in Table 2. The participants will be included if they meet all the following criteria: (1) hospitalized adult patients (≥19 years old) with a primary diagnosis of ADHF; (2) non-valvular atrial fibrillation patients, as documented on electrocardiography, with CHA_2_DS_2_-VASc Score of 2 or more; 3) a diagnosis of HF with reduced EF confirmed by a left ventricular EF of ≤40% using transthoracic echocardiography at the time of admission or within 1 year from the point before admission; and (3) meet at least one of the following criteria: dyspnea at rest, tachypnea (respiratory rate > 20/min), rales, or pulmonary edema on chest X-ray. Participants will be excluded from the study if they have a history of increased bleeding risk (e.g., major surgical procedure or trauma within 30 days; history of major bleeding; clinically significant gastrointestinal bleeding within 180 days; chronic hemorrhagic disorder; intracranial neoplasm, arteriovenous malformation, or aneurysm; platelet count of <90,000/μL), have a contraindication to anticoagulation therapy, have a diagnosis of acute coronary syndrome at the time of admission, are planned for percutaneous coronary intervention, coronary artery bypass graft surgery, or another invasive cardiac intervention (e.g., catheter ablation, pacemaker, cardiac resynchronization therapy, and implantable cardiac defibrillator implantation), are currently on dual antiplatelet therapy (aspirin and adenosine-diphosphate receptor antagonist) or single antiplatelet therapy with a novel antiplatelet agent (e.g., ticagrelor, prasugrel) or warfarin with INR > 2, have cardiogenic shock [systolic blood pressure (SBP): <80 mmHg], creatinine clearance <30 mL/min using creatinine-based Chronic Kidney Disease Epidemiology Collaboration (CKD-EPI) equations, elevated liver enzymes (three-times the upper limit) or liver cirrhosis, uncontrolled hypertension (SBP > 180 mmHg), an allergy, adverse drug reaction, hypersensitivity to rivaroxaban or warfarin, have a life expectancy of <6 months (e.g., metastatic cancer), or are women who are pregnant or of child-bearing age.

**Table 2:** Inclusion and exclusion criteria.

**Inclusion criteria**	**Exclusion criteria**
• 19 years of age or older • Hospitalized patients with a primary diagnosis of ADHF • Non-valvular atrial fibrillation patients, as documented on electrocardiography, with CHA_2_DS_2_-VASc Score of 2 or more • Diagnosis of heart failure with reduced ejection fraction confirmed by left ventricular ejection fraction of ≤40% at the time of the admission or within 1 year from the admission • At least one of the following: i Dyspnea at rest ii Tachypnea (respiratory rate > 20/min) iii Rales iv Pulmonary edema on chest X-ray	• History of increased bleeding risk* • Contraindication to anticoagulation therapy • Acute coronary syndrome diagnosis at the time of the admission • Planned for percutaneous coronary intervention, coronary artery bypass graft surgery, or other cardiac invasive interventions (e.g., catheter ablation, pacemaker, CRT, ICD implantation) • Currently on dual antiplatelet therapy (aspirin and ADP receptor antagonist) or single antiplatelet therapy with a novel antiplatelet agent (e.g., ticagrelor, prasugrel) or warfarin with INR > 2 • Have cardiogenic shock (systolic blood pressure, SBP, < 80 mmHg), • Creatinine clearance < 30 mL/min using creatinine-based CKD-EPI equations • Elevated liver enzymes (3 times the upper normal limit) or liver cirrhosis • Uncontrolled hypertension (SBP > 180 mmHg), • Allergy, adverse drug reaction, or hypersensitivity to rivaroxaban or warfarin • Life expectancy < 6 months (e.g., metastatic cancer) • Women who are pregnant or of child-bearing age

*ADHF, acute decompensated heart failure; CRT, cardiac resynchronization therapy; ICD, implantable cardiac defibrillator implantation; ADP, adenosine-diphosphate; CKD-EPI, Chronic Kidney Disease Epidemiology Collaboration; SBP, systolic blood pressure; INR, international normalized ratio*.

* *Major surgical procedure or trauma within 30 days; history of major bleeding; clinically significant gastrointestinal bleeding within 180 days; chronic hemorrhagic disorder; intracranial neoplasm, arteriovenous malformation, or aneurysm; platelet count <90,000**/**μL*.

### Study Protocol

Eligible patients will be centrally randomized 1:1 to receive either warfarin or rivaroxaban using a study-specific electronic case-report form management system. Patients randomized to the rivaroxaban group will receive rivaroxaban 20 mg orally once daily; those with an estimated glomerular filtration rate of 30–49 mL/min/1.73 m^2^, determined using the Chronic Kidney Disease Epidemiology Collaboration (CKD-EPI) equation, will receive 15 mg. Patients randomized to the warfarin group will receive warfarin 3 mg once daily for two consecutive days (2 mg once daily in patients with the body weight of <50 kg), followed by an appropriate dose once daily prescribed by the investigator at each center according to the patients' prothrombin time. The target INR is 2–3, and low molecular weight heparin (enoxaparin, 1 mg/kg every 12 h) will be administered until the INR of participants reaches 2. All randomized patients will receive standard-of-care for HF management during index hospitalization and follow-up period of 180 days after discharge. Following randomization, HF and any other comorbidities will be managed appropriately. However, anticoagulation with any drugs other than the study treatments will be avoided.

Patients will be assessed periodically at pre-specified study visits during hospitalization [visits 1 (day 1), 2 (day 2), 3 (day 4), and 4 (day 7 or discharge, whichever occurs first)] and after hospitalization [visits 5 (discharge day 30), 6 (discharge day 90), and 7 (discharge day 180)] (Figure 1). Vital signs, and HF signs and symptoms will be assessed according to the New York Heart Association functional class and visual analog scale for dyspnea from discharge to day 180 (Table 3). Additionally, adverse events will be recorded at all visits.

**Table 3:** Assessment schedule.

**Time point**	**In-hospital visits**				**Post-discharge visits**		
	**Visit 1**	**Visit 2**	**Visit 3**	**Visit 4**	**Visit 5**	**Visit 6**	**Visit 7**
	**D1**	**D2**	**D4**	**DC 0 (or D7)**	**DC30 ± 7D**	**DC 90 ± 7D**	**DC 180 ± 7D**
Screening procedures	•						
Medications^*^	•			•			•
Vital signs	•	•	•	•	•	•	•
Physical examination	•	•	•	•	•	•	•
Evaluation of congestion^†^	•	•	•	•	•		•
Height, body weight	•	•	•	•	•	•	•
Prescription of study drug	•			•	•	•	
Laboratory tests (local)	•	•	•	•	•	•	•
Laboratory tests (central)	•	•	•	•	•		•
Human-derived materials	•	•	•	•	•		•
HF symptoms (VAS, NYHA class)	•	•	•	•	•		•
Electrocardiography	•	•	•	•	•	•	•
Chest X-ray	•				•	•	•
Assessment of adverse events	•	•	•	•	•	•	•
Assessment of clinical outcomes	•	•	•	•	•	•	•

*D, day; DC, discharge day; NYHA, New York Heart Association; VAS, visual analog scale*.

* *The following medications taken by a patient within ~24 h prior to screening will be collected: aspirin, clopidogrel, warfarin, angiotensin converting enzyme inhibitor (ACEI), Angiotensin II Receptor Blocker (ARB), beta blocker, ivabradine, entresto, statin, omega 3 fatty acid, furosemide, torsemide, spironolactone, hydrochlorothiazide, thiazide-like, other diuretics, thiazolidinedione (TZD), Dipeptidyl-peptidase 4 (DPP4) inhibitor, metformin, sulfonylurea, Sodium-glucose transport protein 2 (SGLT2) inhibitor, insulin, dronedarone, other anti-arrhythmics. The following concomitant medications at discharge will be collected: aspirin, clopidogrel, warfarin, ACE I, ARB, beta blocker, ivabradine, sacubitril/valsartan, statin, omega-3 polyunsaturated fatty acids, furosemide, torsemide, spironolactone, hydrochlorothiazide, thiazide-like, other diuretics, TZD, DPP4 inhibitor, metformin, sulfonylurea, SGLT2 inhibitor, insulin*.

† *Evaluation parameters are as follows: tachypnea, rales, S3 gallop, edema, orthopnea, bendopnea, neck vein engorgement*.

Blood will be collected locally at pre-specified visits and analyzed for biomarkers and end-organ function. A detailed list of hematology, chemistry, and coagulation assessments is presented in Table 4. Biomarker analyses will be performed in a central laboratory. A subset of blood chemistry tests, including blood urea nitrogen, creatinine, electrolytes (Na^+^, K^+^, Cl^−^), and liver enzymes (serum alanine aminotransferase and aspartate aminotransferase) will be repeated by the central laboratory to confirm the accuracy of the laboratory data from local centers. If there is a discrepancy in blood chemical tests between the central laboratory and local centers, the results from the central laboratory will be used for the analysis. Blood samples will be stored for future biomarker analyses. Electrocardiograms will be obtained and interpreted locally at each visit and sent to a central laboratory for evaluation.

**Table 4:** Hematology, chemistry, and coagulation assessments.

**Category**	**Lists**
Hematology	White blood cell count, red blood cell count, hemoglobin, hematocrit, platelet, mean corpuscular volume, red cell distribution width, neutrophil count, and lymphocyte count
Chemistry	Calcium, inorganic phosphate, fasting glucose, uric acid, cholesterol, total protein, albumin, alkaline phosphatase, total bilirubin, serum alanine aminotransferase, aspartate aminotransferase, sodium, potassium, chloride, blood urea nitrogen, and creatinine
Coagulation	Prothrombin time (INR)
Urine lab	Specific gravity, pH, protein, glucose, ketone, red blood cell, urobilinogen, bilirubin, nitrite, and white blood cell

*INR, international normalized ratio*.

### Statistical Considerations

#### Sample Size

Data on hsTn in patients with ADHF and AF after anticoagulation treatment are lacking. However, hsTn was studied as a potential biomarker in patients with ADHF in the RELAX-AHF trial of serelaxin (18, 19). As the hsTn level is related to ADHF, information on hsTn from RELAX-AHF can be used, based on the following assumptions. Based on a feasibility assessment, 150 patients (75 patients in each treatment group) are planned for enrolment. In the RELAX-AHF study, the geometric mean of hsTn at the baseline and 95% confidence interval (CI) was 0.034 (0.032–0.037), with 581 patients. Therefore, assuming the log-scaled standard deviation is 0.75, with a sample size of 75 patients, the maximum imprecision of the geometric mean is 18%, which is considered to provide reasonable precision of estimate. The rate of drop-out is not considered in the calculation as the primary endpoints will be evaluated during hospitalization, and the drop-out rate is anticipated to be very low.

#### Analyses

As the current study is not powered for clinical hypothesis testing, statistical analyses will be explorative and descriptive. All variables will be analyzed descriptively: categorical variables will be presented as frequency tables (absolute and relative frequencies with 95% CI) and continuous variables by summary statistics (mean, standard variation, minimum, median quartile, and maximum, and 95% CI). We will analyze circulating hsTn and other quantitative biomarkers with natural log-transformation, and the geometric mean with 95% CI will be provided for each visit. The adjusted geometric means of the maximum change in hsTn from the baseline to hospitalization will be evaluated using an analysis of covariance model, with the value at admission as a covariate, and stratification factors used for randomization and treatment as a factor. The treatment geometric mean ratio, which reflects the treatment difference between rivaroxaban and warfarin, as well as its 95% CI, will also be provided.

A survival analysis will be performed to describe the time from hospital admission to the composite endpoint, including all-cause mortality and rehospitalization. Kaplan–Meier estimates and plots will be obtained for each treatment. The hazard ratio for rivaroxaban over warfarin and its 95% CI will be generated from the Cox-proportional model. The incidence of all-cause mortality during hospitalization will be summarized in a frequency table with the corresponding percentage and 95% CI. The incidence of major and minor bleeding events during the study will be summarized in a frequency table with percentage and corresponding 95% CI. To adjust variations in treatment duration during the study, the incidence rate (number of patients with bleeding events divided by the cumulative person-time on treatment) will also be provided.

## Discussion

Despite advances in pharmacological therapy and devices for patients with HF, mortality, both in-hospital and post-discharge, remains high. Ongoing myocardial ischemic damage and hypercoagulability during and after hospitalization might be associated with poor outcomes. A recent prospective trial demonstrated that treatment with low-dose rivaroxaban, a factor Xa antagonist, decreased the rate of stroke or TIA in patients with chronic HF and coronary artery disease with sinus rhythm after a recent worsening episode compared with the placebo (10). This suggests that anticoagulation with factor Xa antagonists might reduce myocardial damage during hospitalization and improve outcomes in patients with ADHF and AF compared with warfarin treatment. ROAD HF-AF is designed to evaluate the potential benefits of rivaroxaban on myocardial and renal damage compared with VKAs, using surrogate biomarkers in hospitalized patients with ADHF and AF.

### Early Effect of Rivaroxaban on Circulating hsTn Levels in Hospitalized Patients With Acute Decompensated HF

Troponin (Tn) level is frequently elevated in patients with ADHF, even without clinically evident coronary artery disease (20). The circulating troponin (cTn) level has been reported to be a reliable prognostic marker for short- and long-term outcomes in patients with ADHF (21, 22). Furthermore, studies on serial cTn measurements throughout hospitalization reported that patients with persistently elevated cTn had a worse prognosis than those without persistently elevated cTn (23). More recently, in a subgroup analysis of the RELAX-AHF study (19), the peak change in hsTn was found to be an independent risk predictor for cardiovascular death or renal injury/HF hospitalization and cardiovascular mortality up to 6 months. We will evaluate the benefit of rivaroxaban compared with VKAs using the peak change in hsTn in patients with ADHF and AF during hospitalization. According to RELAX-AHF study, the peak value of high hsTn was measured at day 2–day 5. Therefore, measuring the maximum change in the hsTn level over 72 h from admission as a primary endpoint would be reasonable.

### Justification for the Extended Therapeutic Intervention and Follow-Up

Studies on the efficacy of novel target drugs, such as ularitide or serelaxin, during short-term hospitalization reported unsatisfactory long-term outcomes in patients with ADHF (24, 25). Furthermore, the results of the ASTRONAUT study, which investigated the change in Tn between the pre- and early post-discharge periods (1 month) in hospitalized patients with ADHF, demonstrated that patients experienced an extended period of vulnerability to cardiac injury after the index hospitalization (26). Therefore, in our study, anticoagulation evaluation in both rivaroxaban and VKAs groups will be continued for 6 months, and the long-term effect will be assessed as a secondary outcome. For example, the serial changes in hsTn and clinical outcomes will be followed up to 6 months. The extended treatment and follow-up assessments are expected to elucidate the long-term efficacy and safety of rivaroxaban. Furthermore, we will evaluate the clinical effect of changes in hsTn during hospitalization and early-discharge on long-term clinical outcomes.

### Thrombogenicity Biomarkers as a Secondary Outcome

In addition to hsTn, we will investigate changes in novel biomarkers associated with thrombogenicity, renal comorbidity, and cardiac fibrosis. In terms of thrombogenicity biomarkers, we will explore changes in D-dimer, TAT complex, and PAI-1 as a secondary outcome. D-Dimer is a product of fibrin turnover and is widely utilized as a biomarker of thrombosis. Patients with HF were reported to have increased levels of D-dimer and TAT complex; elevated levels of D-dimer at the time of hospital admission have been associated with in-hospital stroke risk in patients with HF (27). The TAT complex is a protein complex comprising inhibited thrombin with antithrombin and reflects the functional state of the coagulation system (28). Additionally, PAI-1 inhibits the formation of plasmin and breakdown of fibrin clots and is thus a crucial inhibitor of the fibrinolytic pathway (29). Elevated PAI-1 levels are independently associated with the risk of cardiovascular disease (30). In our study, an analysis of these biomarkers will provide valuable information on the effects of rivaroxaban and warfarin on thrombosis.

### Renal Biomarkers as a Secondary Outcome

Here, we will explore changes in NGAL and NAG, which are markers of renal injury, from the baseline to the day of discharge, and 30 and 180 days after discharge to elucidate the benefits of rivaroxaban compared with a VKA. NGAL, a protein bound to gelatinase, was initially detected in neutrophil granules (11). Given that NGAL is secreted in the thick ascending limb of the kidney in response to renal injury, it has been used as a biomarker for the early detection of renal injury in various clinical settings. Furthermore, NAG is an enzyme found in the lysosomes of renal proximal tubular cells. Urinary NAG excretion is increased under proximal tubular cell injury. Several studies have shown that increased urinary NAG is independently related to adverse outcomes in patients with acute and chronic HF (31). Thus, in our study, assessing changes in these renal biomarkers will provide valuable information regarding the renal benefits of rivaroxaban compared with the VKA in conjunction with other established biomarkers of renal function.

### Cardiac Fibrosis Biomarkers as a Secondary Outcome

sST2 is a soluble isoform of ST2, which is released under myocardial stress. sST2 can be used as a useful prognostic marker in patients with chronic HF, and a recent study reported the usefulness of serial sST2 measurements in the ADHF setting. We will also investigate galectin-3, which is associated with inflammation and fibrosis. In the PRIDE study (32), galectin-3 level had an independent and incremental prognostic value over NT-proBNP for predicting mortality and recurrent HF in patients with ADHF. The use of sST2 and galectin-3 as prognostic biomarkers was recommended in the 2017 American College of Cardiology/American Heart Association/Heart Failure Society of America HF guidelines (33). Considering that myocardial fibrosis is a chronic process, we will evaluate changes in these surrogates for cardiac fibrosis from admission to 6 months after discharge.

## Limitations

This is an exploratory, descriptive study, which will include 150 patients. Therefore, it is not powered to determine differences in clinical adverse events, including mortality or major/minor bleeding. However, this study will provide information on the potential beneficial effect of rivaroxaban compared to warfarin on myocardial and renal injury by the biomarker levels. This information is expected to be used as substantial evidence for future large-scale clinical studies. Additionally, this study is not blinded, which can lead to potential bias.

## Conclusions

We hypothesize that, compared with warfarin, rivaroxaban will reduce myocardial/renal injury and hemodynamic stress as reflected by the biomarker levels with an onset within 72 h, with an acceptable tolerability in hospitalized patients with ADHF and AF. Based on the results of this study, we hope to facilitate future biomarker-based, large-scale outcome trials in patients with ADHF and AF.

## Ethics Statement

The studies involving human participants were reviewed and approved by the Institutional Review Board of each participating center: Severance Hospital (No. 4-2017-0776), Keimyung University Dongsan Medical Center (2017-11-070), Kyung Hee University Hospital (KHUH 2017-12-010-002), Yeungnam University Medical Center (2017-11-027), Chung-Ang University Medical Center (1711-010-304), Yonsei University Wonju Severance Christian Hospital (CR117080), Gangnam Severance Hospital (3-2017-0287), Gachon University Gil Medical Center (GAIRB2018-063), Ewha Womans University Hospital (2017-11-032), and Catholic University of Korea Bucheon ST. MARY‘S Hospital (HC18-MEDV-0015). The patients/participants provided their written informed consent to participate in this study.

## Author Contributions

IC, JO, and S-MK organized the database. IC and JO performed the statistical analysis. IC wrote the first draft of the manuscript. All authors contributed to the conception, design, patient enrollment of the study, contributed to manuscript revision, read, and approved the submitted version.

## Funding

This work was supported by Bayer Pharma, Berlin, Germany.

## Conflict of Interest

S-MK reports grants and consultant fees from Bayer Pharma. The remaining authors declare that the research was conducted in the absence of any commercial or financial relationships that could be construed as a potential conflict of interest.

## Publisher's Note

All claims expressed in this article are solely those of the authors and do not necessarily represent those of their affiliated organizations, or those of the publisher, the editors and the reviewers. Any product that may be evaluated in this article, or claim that may be made by its manufacturer, is not guaranteed or endorsed by the publisher.

## References

[1] Ponikowski P Voors AA Anker SD Bueno H Cleland JGF Coats AJS . 2016 ESC guidelines for the diagnosis treatment of acute chronic heart failure: the task force for the diagnosis treatment of acute chronic heart failure of the european society of cardiology (ESC) developed with the special contribution of the heart failure association (HFA) of the ESC. Eur Heart J. (2016) 37:2129–200. 10.1093/eurheartj/ehw128 27206819

[2] Kang S-M Cho M-C. Prognostic factors in hospitalization for heart failure in Asia. Heart Fail Clin. (2015) 11:543–50. 10.1016/j.hfc.2015.07.006 26462094

[3] Witt BJ Brown RD Jacobsen SJ Weston SA Ballman KV Meverden RA . Ischemic stroke after heart failure: a community-based study. Am Heart J. (2006) 152:102–9. 10.1016/j.ahj.2005.10.018 16824838

[4] Nieminen MS Brutsaert D Dickstein K Drexler H Follath F Harjola V-P . EuroHeart failure survey II (EHFS II): a survey on hospitalized acute heart failure patients: description of population. Euro Heart J. (2006) 27:2725–36. 10.1093/eurheartj/ehl193 17000631

[5] Kirchhof P Benussi S Kotecha D Ahlsson A Atar D Casadei B . 2016 ESC Guidelines for the management of atrial fibrillation developed in collaboration with EACTS. Eur Heart J. (2016) 37:2893–962. 10.5603/KP.2016.0172 27567408

[6] Ferreira J Ezekowitz MD Connolly SJ Brueckmann M Fraessdorf M Reilly PA . Dabigatran compared with warfarin in patients with atrial fibrillation and symptomatic heart failure: a subgroup analysis of the RE-LY trial. Eur J Heart Fail. (2013) 15:1053–61. 10.1093/eurjhf/hft111 23843099

[7] Diepen Sv Hellkamp AS Patel MR Becker RC Breithardt G Hacke W . Efficacy and safety of rivaroxaban in patients with heart failure and nonvalvular atrial fibrillation. Circul Heart Fail. (2013) 6:740–7. 10.1161/CIRCHEARTFAILURE.113.000212 23723250

[8] Zannad F Stough WG Regnault V Gheorghiade M Deliargyris E Gibson CM . Is thrombosis a contributor to heart failure pathophysiology? Possible mechanisms, therapeutic opportunities, and clinical investigation challenges. Int J Cardiol. (2013) 167:1772–82. 10.1016/j.ijcard.2012.12.018 23298559

[9] Gheorghiade M Vaduganathan M Fonarow GC Greene SJ Greenberg BH Liu PP . Anticoagulation in heart failure: current status and future direction. Heart Fail Rev. (2013) 18:797–813. 10.1007/s10741-012-9343-x 22987320

[10] Mehra MR Vaduganathan M Fu M Ferreira JP Anker SD Cleland JGF . A comprehensive analysis of the effects of rivaroxaban on stroke or transient ischaemic attack in patients with heart failure, coronary artery disease, and sinus rhythm: the COMMANDER HF trial. Euro Heart J. (2019) 40:3593–602. 10.1093/eurheartj/ehz427 31461239 PMC6868495

[11] Ibrahim NE Januzzi JL Jr. Established and emerging roles of biomarkers in heart failure. Circ Res. (2018) 123:614–29. 10.1161/CIRCRESAHA.118.312706 30355136

[12] Collins SP Jenkins CA Harrell FE Jr Liu D Miller KF Lindsell CJ . Identification of emergency department patients with acute heart failure at low risk for 30-day adverse events: the STRATIFY decision tool. JACC Heart Fail. (2015) 3:737–47. 10.1016/j.jchf.2015.05.007 26449993 PMC4625834

[13] Takashio S Yamamuro M Izumiya Y Sugiyama S Kojima S Yamamoto E . Coronary microvascular dysfunction and diastolic load correlate with cardiac troponin T release measured by a highly sensitive assay in patients with nonischemic heart failure. J Am Coll Cardiol. (2013) 62:632–40. 10.1016/j.jacc.2013.03.065 23644085

[14] Metra M Cotter G Davison BA Felker GM Filippatos G Greenberg BH . Effect of serelaxin on cardiac, renal, and hepatic biomarkers in the relaxin in acute heart failure (RELAX-AHF) development program: correlation with outcomes. J Am Coll Cardiol. (2013) 61:196–206. 10.1016/j.jacc.2012.11.005 23273292

[15] Masson S Anand I Favero C Barlera S Vago T Bertocchi F . Serial measurement of cardiac troponin T using a highly sensitive assay in patients with chronic heart failure: data from 2 large randomized clinical trials. Circulation. (2012) 125:280–8. 10.1161/CIRCULATIONAHA.111.044149 22139751

[16] Schulman S Kearon C Subcommittee Subcommittee on Control of Anticoagulation of the Scientific and Standardization Committee of the International Society on Thrombosis and Haemostasis. Definition of major bleeding in clinical investigations of antihemostatic medicinal products in non-surgical patients. J Thromb Haemost. (2005) 3:692–4. 10.1111/j.1538-7836.2005.01204.x 15842354

[17] Kjoller E Hilden J Winkel P Frandsen NJ Galatius S Jensen G . Good interobserver agreement was attainable on outcome adjudication in patients with stable coronary heart disease. J Clin Epidemiol. (2012) 65:444–53. 10.1016/j.jclinepi.2011.09.011 22257841

[18] Teerlink JR Cotter G Davison BA Felker GM Filippatos G Greenberg BH . Serelaxin, recombinant human relaxin-2, for treatment of acute heart failure (RELAX-AHF): a randomised, placebo-controlled trial. Lancet. (2013) 381:29–39. 10.1016/S0140-6736(12)61855-8 23141816

[19] Felker GM Mentz RJ Teerlink JR Voors AA Pang PS Ponikowski P . Serial high sensitivity cardiac troponin T measurement in acute heart failure: insights from the RELAX-AHF study. Eur J Heart Fail. (2015) 17:1262–70. 10.1002/ejhf.341 26333655

[20] Kociol RD Pang PS Gheorghiade M Fonarow GC O'Connor CM Felker GM. Troponin elevation in heart failure: prevalence, mechanisms, and clinical implications. J Am Coll Cardiol. (2010) 56:1071–8. 10.1016/j.jacc.2010.06.016 20863950

[21] Peacock WF De Marco T Fonarow GC Diercks D Wynne J Apple FS . Cardiac troponin and outcome in acute heart failure. N Engl J Med. (2008) 358:2117–26. 10.1056/NEJMoa0706824 18480204

[22] Parenti N Bartolacci S Carle F Angelo F. Cardiac troponin I as prognostic marker in heart failure patients discharged from emergency department. Intern Emerg Med. (2008) 3:43–7. 10.1007/s11739-008-0092-8 18273567

[23] Metra M Nodari S Parrinello G Specchia C Brentana L Rocca P . The role of plasma biomarkers in acute heart failure. Serial changes and independent prognostic value of NT-proBNP and cardiac troponin-T. Eur J Heart Fail. (2007) 9:776–86. 10.1016/j.ejheart.2007.05.007 17573240

[24] Packer M O'Connor C McMurray JJV Wittes J Abraham WT Anker SD . Effect of ularitide on cardiovascular mortality in acute heart failure. N Engl J Med. (2017) 376:1956–64. 10.1056/NEJMoa1601895 28402745

[25] Metra M Teerlink JR Cotter G Davison BA Felker GM Filippatos G . Effects of serelaxin in patients with acute heart failure. N Engl J Med. (2019) 381:716–26. 31433919 10.1056/NEJMoa1801291

[26] Greene SJ Butler J Fonarow GC Subacius HP Ambrosy AP Vaduganathan M . Pre-discharge and early post-discharge troponin elevation among patients hospitalized for heart failure with reduced ejection fraction: findings from the ASTRONAUT trial. Eur J Heart Fail. (2018) 20:281–91. 10.1002/ejhf.1019 29044915 PMC6429915

[27] Hamatani Y Nagai T Nakai M Nishimura K Honda Y Nakano H . Elevated plasma D-dimer level is associated with short-term risk of ischemic stroke in patients with acute heart failure. Stroke. (2018) 49:1737–40. 10.1161/STROKEAHA.118.021899 29880555

[28] Lippi G Cervellin G Franchini M Favaloro EJ. Biochemical markers for the diagnosis of venous thromboembolism: the past, present and future. J Thromb Thrombolysis. (2010) 30:459–71. 10.1007/s11239-010-0460-x 20213258

[29] Song C Burgess S Eicher JD O'Donnell CJ Johnson AD. Causal effect of plasminogen activator inhibitor type 1 on coronary heart disease. J Am Heart Assoc. (2017) 6:e004918. 28550093 10.1161/JAHA.116.004918 PMC5669150

[30] Jung RG Motazedian P Ramirez FD Simard T Di Santo P Visintini S . Association between plasminogen activator inhibitor-1 and cardiovascular events: a systematic review and meta-analysis. Thromb J. (2018) 16:12. 10.1186/s12959-018-0166-4 29991926 PMC5987541

[31] Jungbauer CG Birner C Jung B Buchner S Lubnow M von Bary C . Kidney injury molecule-1 and N-acetyl-beta-D-glucosaminidase in chronic heart failure: possible biomarkers of cardiorenal syndrome. Eur J Heart Fail. (2011) 13:1104–10. 10.1093/eurjhf/hfr102 21846754

[32] van Kimmenade RR Januzzi JL Ellinor PT Sharma UC Bakker JA Low AF . Utility of amino-terminal pro-brain natriuretic peptide, galectin-3, and apelin for the evaluation of patients with acute heart failure. J Am Coll Cardiol. (2006) 48:1217–24. 10.1016/j.jacc.2006.03.061 16979009

[33] Yancy CW Jessup M Bozkurt B Butler J Casey DE Colvin MM . 2017 ACC/AHA/HFSA focused update of the 2013 ACCF/AHA guideline for the management of heart failure: a report of the American college of cardiology/American heart association task force on clinical practice guidelines and the heart failure society of America. J Card Fail. (2017) 23:628–51. 10.1161/CIR.0000000000000509 28461259

